# COVID-19- Experiences and support needs of children and young people with Hydrocephalus and parents in the United Kingdom

**DOI:** 10.1007/s00381-023-05980-7

**Published:** 2023-05-20

**Authors:** Nicole Collaço, Anna Campion, Roisin McNicholas, Anne-Sophie Darlington

**Affiliations:** 1 School of Health Sciences, Centre for Psychosocial Research in Cancer (CentRIC+), University of Southampton, Southampton, SO17 1BJ England; 2 Southampton General Hospital, Southampton, England; 3 School of Medicine, University of Leeds, Leeds, England

**Keywords:** Cerebrospinal fluid, Mental health, Well-being, Worries

## Abstract

**Purpose:**

Little is known about the impact of COVID-19 on children and young people (CYP) with hydrocephalus and their families. This study explored the experiences and support needs of CYP with hydrocephalus and parents who have a child with hydrocephalus during the COVID-19 pandemic.

**Methods:**

CYP with hydrocephalus and parents of CYP with hydrocephalus in the United Kingdom completed an online survey with open and closed questions exploring experiences, information, support needs and decision making processes. Qualitative thematic content analysis and descriptive quantitative analyses were undertaken.

**Results:**

CYP aged 12-32 years (n=25) and parents of CYP aged 0-20 years (n=69) responded. Parents (63.5%) and CYP (40.9%) worried about the virus, and both were vigilant for virus symptoms (86.5% and 57.1%). Parents (71.2%) and CYP (59.1%) worried about their child/feeling more isolated during the virus outbreak. Parents felt concerned about having to take their child to hospital with a suspected shunt problem during the virus outbreak (64.0%). Qualitative findings reported the following themes: (1) Healthcare and treatment provision: delays and challenges to access and availability of care (2) Impact of COVID-19/lockdown on daily lives and routines, and (3) Provision of information and support for parents and CYP with hydrocephalus.

**Conclusion:**

The impact of COVID-19 and national measures to control the spread of the virus- no contact with anyone outside the household significantly impacted the daily lives and routines of CYP with hydrocephalus and parents. Social engagements were missed, families faced challenges to their work life, education and access to health care and support, which subsequently contributed negatively to their mental wellbeing. CYP and parents highlighted a need for clear, timely and targeted information to address their concerns.

**Supplementary Information:**

The online version contains supplementary material available at 10.1007/s00381-023-05980-7.

## Introduction

The World Health Organisation (WHO) declared the outbreak of novel severe acute respiratory syndrome coronavirus 2 (SARS-CoV-2), known as ‘COVID-19’ a global pandemic in March 2020 [1]. In the United Kingdom (UK), preventative measures were implemented to slow the rate of infection and limit its spread through a national ‘lockdown’, which saw the closure of schools, workplaces and restrictions placed on outdoor movements, such as exercise [2–4]. The National Health Service (NHS) in the United Kingdom (UK) was forced to suspend many services which resulted in a significant decrease in the number of outpatient appointments and elective procedures taking place [5]. Globally, significant adjustments were made within neurosurgical practice with respect to clinics and surgical planning [6].

COVID-19 cases are lower in children and young people (CYP) [7], and their symptoms are less severe [8]. Despite this, the pandemic has had a considerable impact on the mental and physical wellbeing of CYP with serious medical conditions and their parents [4, 8, 9].

Hydrocephalus is one of the most common conditions treated by paediatric neurosurgeons, and is characterised by an excess accumulation of cerebrospinal fluid (CSF) within the cerebral ventricles, ventricular dilatation, and raised intracranial pressure[10, 11]. Paediatric hydrocephalus can arise idiopathically, or as a result of congenital or acquired conditions such as spina bifida, infection and haemorrhage [11, 12]. It is associated with a range of neurological conditions such as epilepsy and learning disability [12]. Treatment consists of CSF drainage with a shunt or endoscopic third ventriculostomy (ETV) [11, 13]. Whilst effective, the burden of hydrocephalus treatment on families and healthcare systems is considerable, with many children requiring frequent readmissions [14], and around 40% of primary shunts failing within 2 years of placement [15].

Prior to the pandemic, studies have identified elevated psychosocial issues within this population, including high levels of behavioural difficulties and substantially lower quality of life [12, 16]. Given the complexity of this condition [17], the pre-existing psychosocial difficulties within this population, and the known impact of the pandemic on CYP with chronic medical conditions and their parents, CYP with hydrocephalus and their parents are at an elevated risk for psychosocial difficulties and lower quality of life during the pandemic [17]. The aim of the present study was to explore the pandemic’s impact on CYP who have hydrocephalus and parents who have a child with hydrocephalus.

## Methods

This study surveyed parents of a child with hydrocephalus and CYP with hydrocephalus. The online survey assessed experiences, information, decision making and support needs. This study is part of a larger longitudinal study (the SHARE study) which assessed experiences of parents and CYP across different conditions during the COVID-19 pandemic[3, 4, 9, 18, 19]. The hydrocephalus survey opened on 13 May 2020 and closed on 10 August 2020, thus capturing the experiences and needs of these respondents during easing of lockdown restrictions of the COVID-19 pandemic within the UK. The study was approved by the University of Southampton and UK NHS Health Research Authority Research Ethics Committees (IRAS nr. 282176).

### Participants

Parents of a child with hydrocephalus, and CYP aged between 10 and 39 years, and able to read and respond in English were eligible. Respondents were recruited through social media, and two national hydrocephalus charities (Harry’s Hydrocephalus Awareness Trust and SHINE). Electronic consent was obtained before completing the online survey.

### Survey

The survey content was informed through literature [20–22], clinician, patient and parental input. Feedback from a hydrocephalus charity, and parents in our parent/patient involvement group identified changes to be made regarding content, phrasing and completeness. The survey contained the following sections and a number of open and closed item statements in each section: Experience, Information, Decisions, and Support needs. The response options for the closed statement items included: Not at all, A little, Quite a bit, Very much (except for two conditional questions: Yes/No as response options). The beginning of each section included a free text box for comments, for example in the Experiences section *‘Can you tell us about your experiences and views on the virus in relation to your child with Hydrocephalus?’; Information: ‘Can you tell us where you get information on the virus and what other information you might need?’*

### Data analysis

Descriptive statistics using IBM Statistical Package for Social Science (SPSS) was conducted to summarize demographic information and closed statement items. A binary outcome was created from analysing closed statement items through collapsing the lowest two response options- Not at all, A little, and the highest two responses- Quite a bit, Very much.

Thematic content analysis was conducted on the open text box data [23, 24]. Firstly, comments were openly coded into broad categories by two researchers (NC and RM), which informed an initial coding framework. Secondly the framework was then used to categorise all comments from the survey, which then underwent further refinement. Lastly overarching themes were developed from the analysis of similarities in the content across categories. The number of comments were counted to identify weight of themes. Due to overlap in comments to categories, the total number of comments did not match the number of participants.

## Results

Sixty-nine parents responded to the survey of which 87.0% (n=60) were mothers, 4.3% (n=3) were fathers, 4.3% (n=3) were either a grandparent/foster carer/adoptive parent, 4.3% (n=3) was missing data. The median age of CYP of responding parents was 8.5 years. The majority of respondents resided in the UK 91.3% (n= 63).

Twenty-five CYP responded to the survey and the median age of CYP completing the survey was 22 years old. Respondents were located in the UK, 100.0% (n=25). Table 1 summarizes respondents’ characteristics.

**Table 1:** Respondent characteristics

**Surveys**	**Children and Young People (CYP)**	**Parents**
**Completed by, n**	25	69
**Participant age, years, median (range)**	22 (12-32)	38 (23-60)
**Child’s age, years, median (range)**		8.5 (0-20)
**Gender**		(of parents child)
Female Male Missing	23 (92.0%) 2 (8.0%) 0 (0.0%)	31 (44.9%) 36 (52.2%) 2 (2.9%)
**Relationship to CYP**		
Mother Father Foster Carer Adoptive Parent Grandparent Missing		60 (87.0%) 3 (4.3%) 1 (1.4%) 1 (1.4%) 1 (1.4%) 3 (4.3%)
**Respondents by nation, n (%)**		
United Kingdom Ireland Missing	25 (100.0%) 0 (0.0%) 0 (0.0%)	63 (91.3%) 4 (5.8%) 2 (2.9%)
**Cause of Hydrocephalus, n (%)**		
Infection (e.g. meningitis, encephalitis) Aqueductal stenosis Arachnoid cyst Chiari malformation Head injury Intraventricular haemorrhage associated with premature birth Other Haemorrhage (e.g. subarachnoid, cerebellar) Dandy walker malformation Unsure/Unknown Other Missing	2 (8.0%) 3 (12.0%) 0 (0.0%) 2 (8.0%) 1 (4.0%) 3 (12.0%) 3 (12.0%) 1 (4.0%) 5 (20.0%) 4 (16.0%) 1 (4.0%)	4 (5.8%) 6 (8.7%) 4 (5.8%) 8 (11.6%) 1 (1.4%) 12 (17.4%) (2, 2.9%) 0 (0.0%) 7 (10.1%) 23 (33.3%) 2 (2.9%)
**Had a permanent shunt, n (%)**		(parents child)
Yes No Unsure Missing	21 (84.0%) 3 (12.0%) 1 (4.0%) 0 (0.0%)	61 (88.4%) 5 (7.2%) 1 (1.4%) 2 (2.9%)
**Has a permanent shunt now, n (%)**		(parents child)
Yes Missing	19 (76.0%) 6 (24.0%)	57 (82.6%) 12 (14.5%)
**Most recent shunt operation, n (%)**		
2000 2003 2005 2006 2007 2008 2009 2010 2011 2012 2013 2014 2015 2016 2017 2018 2019 2020 Missing	1 (4.0%) 0 (0.0%) 1 (4.0%) 3 (12.0%) 1 (4.0%) 1 (4.0%) 1 (4.0%) 1 (4.0%) 0 (0.0%) 2 (8.0%) 0 (0.0%) 2 (8.0%) 0 (0.0%) 1 (4.0%) 0 (0.0%) 2 (8.0%) 2 (8.0%) 2 (8.0%) 5 (20.0%)	0 (0.0%) 1 (1.4%) 0 (0.0%) 1 (1.4%) 1 (1.4%) 1 (1.4%) 2 (2.9%) 3 (4.3%) 1 (1.4%) 2 (2.9%) 1 (1.4%) 1 (1.4%) 2 (2.9%) 7 (10.1%) 6 (8.7%) 9 (13.0%) 13 (18.8%) 8 (11.6%) 10 (14.5%)
**Had other hydrocephalus related operations or procedures, n (%)**		(parents child)
Yes No Unsure Missing	16 (64.0%) 8 (32.0%) 1 (4.0%) 0 (0.0%)	37 (53.6%) 30 (43.5%) 0 (0.0%) 2 (2.9%)

**Fig. 1:**
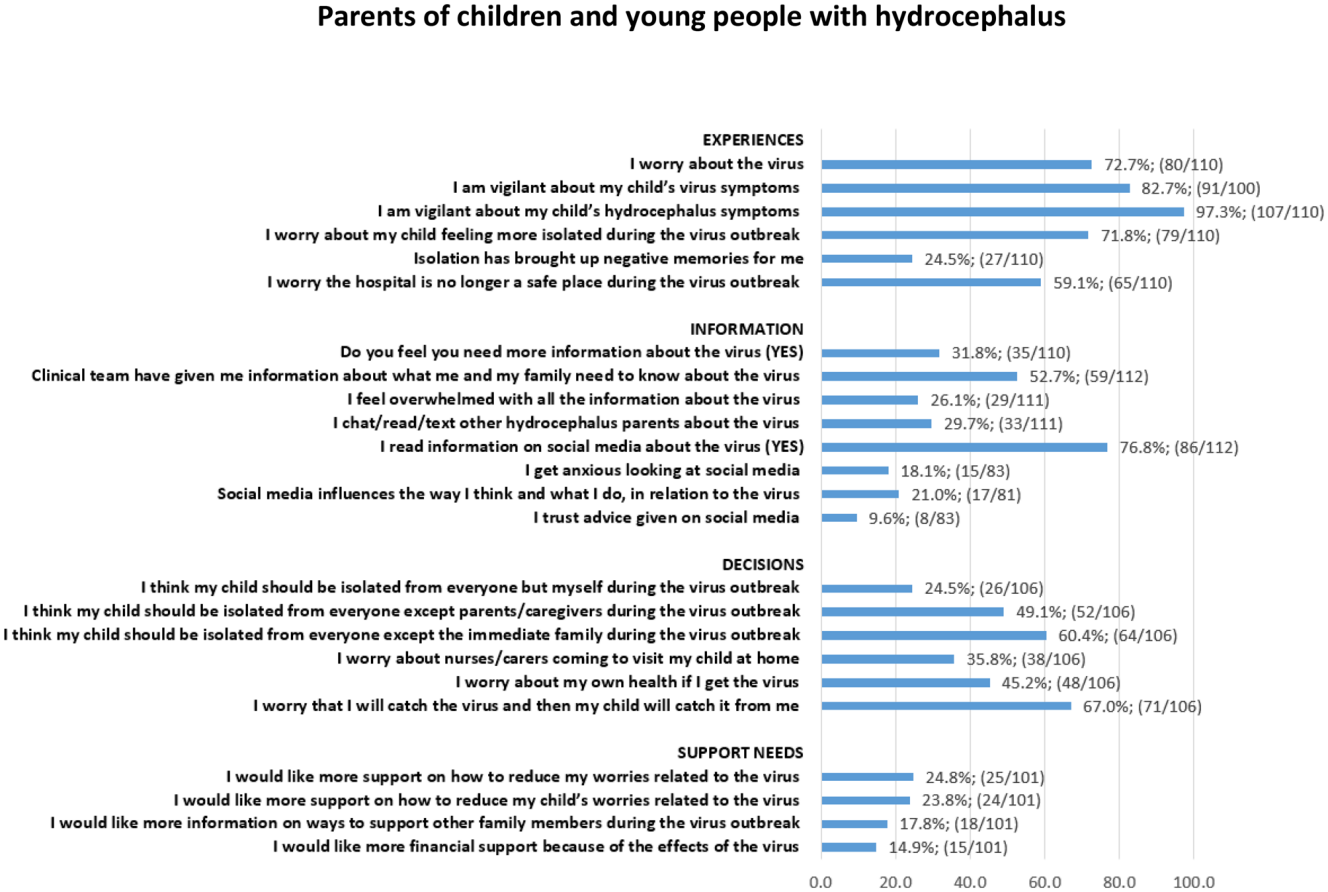
Closed statement items from parent survey

**Fig. 2:**
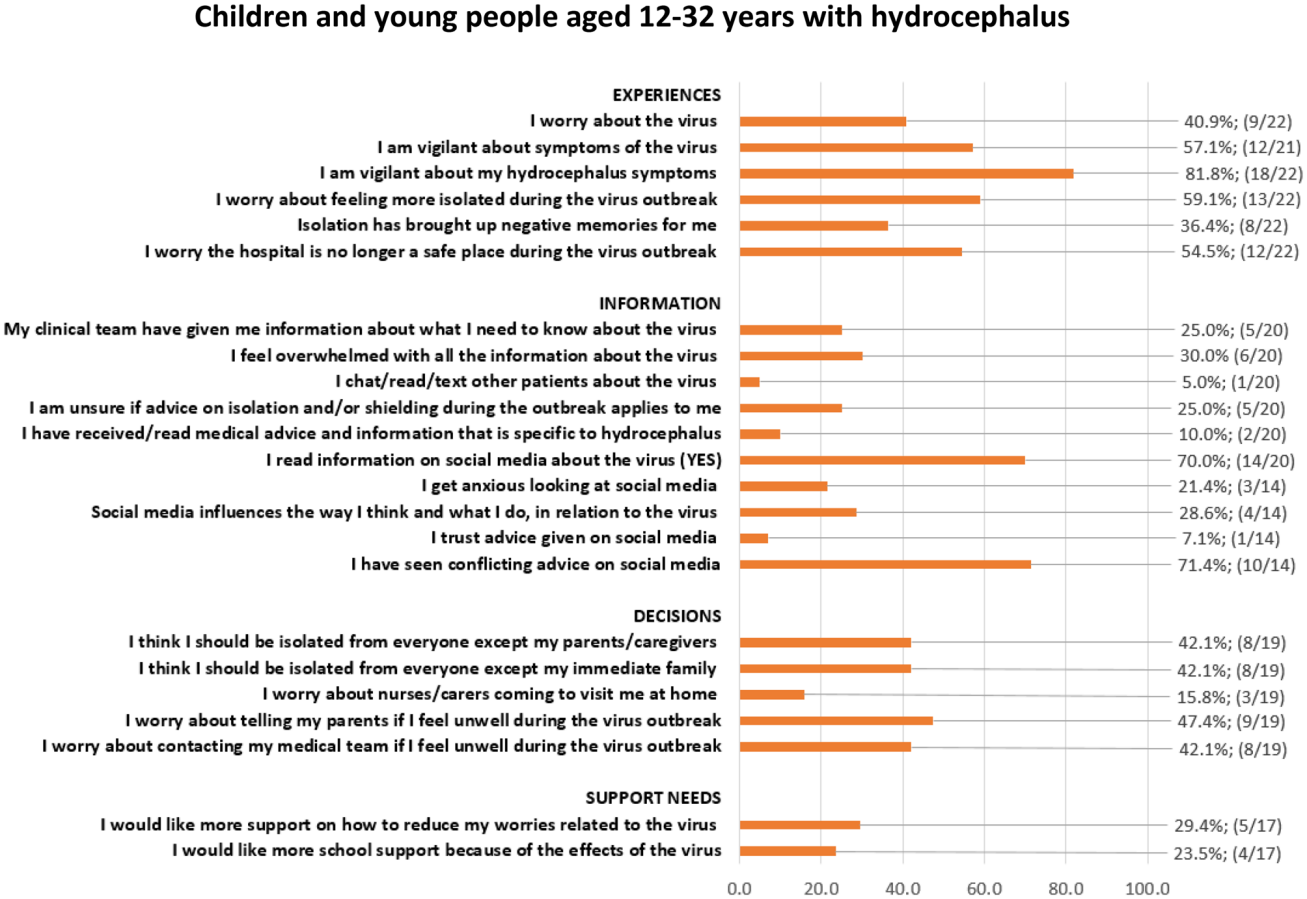
Closed statement items from children and young people survey

Quantitative results from the closed statement items are presented in the figures below for parents (Fig. 1) and CYP (Fig. 2) as numbers and percentage of the total that responded ‘Quite a bit’ and ‘Very much’ to each statement.

### Parent survey

Parents worry about the virus (63.5%) and were vigilant for their child’s virus symptoms or hydrocephalus symptoms triggered by the virus (86.5%, 90.4% respectively). They reported feeling worried about their child feeling more isolated during the virus outbreak (71.2%) and some reported that isolation had bought up negative memories for them (26.9%). Just over half of respondents were concerned that the hospital was no longer a safe place (51.9%), with many worrying that their child’s surgeries and/or clinic appointments would be postponed or cancelled during the virus outbreak (59.6%). A large number of parents worried that they wouldn’t be able to visit their child in hospital during the virus outbreak (69.2%). A small proportion of parents reported receiving information from their child’s clinical team about the virus (17.0%), with an even lower number receiving/reading medical advice and information that was specific to hydrocephalus (15.1%). The majority of parents read information about the virus on social media (84.9%), however a large proportion reported the advice they’d seen to be conflicting (62.8%) and only a small minority of parents reported being influenced by social media in relation to what they do and think about the virus (6.8%) or trusting the advice given on social media (4.5%). Many parents felt worried that they would catch the virus and pass it onto their child (60.0%), and a significant number felt concerned about having to take their child to hospital with a suspected shunt problem during the virus outbreak (64.0%).

### Children and young people survey

CYP were also worried about the virus (40.9%). CYP were vigilant about symptoms of the virus (57.1%) and its potential impact on their hydrocephalus symptoms (81.8%). They worried about feeling more isolated during the virus outbreak (59.1%) and that the hospital was no longer a safe place (54.5%). The majority of CYP read information on social media about the virus (70.0%), although they reported that this information was conflicting (71.4%). Some CYP reported that social media influences the way they think and what they do in relation to the virus (28.6%), although a small number of CYP reported trusting the advice given on social media (7.1%). Just under half of respondents reported feeling worried about telling their parents if they felt unwell during the virus outbreak (47.4%).

### Open text boxes

Responses to open questions contained a total of 278 number of quotes (72 Experiences, 71 Information, 69 Decision making and 66 Support—totalled from CYP and parent responses). Parents (72.4%) and CYP (84.0%) completed the ‘Experiences’ open text box and between 70% and 80% completed the other open text boxes. Respondents appeared to repeat responses from the Experiences open text box in subsequent open text boxes. To avoid replication of findings, the themes from the ‘Experiences’ open text box are described below and presented with additional quotes in Table S2 (Online Resource 1).

Three main themes were identified: Healthcare and treatment provision: delays and challenges to access and availability of care, Impact of COVID-19/lockdown on daily lives and routines and Provision of information and support for parents and children and young people with hydrocephalus.

### Healthcare and treatment provision: restrictions, delays and challenges to access and availability of care

Parents reported that COVID-19 caused disruptions to their child’s treatment and access to healthcare provision. Some parents (n=4) reported that their child’s test results and treatment were delayed or that their appointments/therapies were cancelled (n=9). A minority of parents expressed concern about access to hospital services due to COVID-19 (n=3) and both CYP (n=4) and parents (n=3) worried about requiring/their child requiring shunt surgery during the pandemic due to their concerns about the availability of care. Parents (n=11) and CYP also reported concerns about visiting/staying in hospitals overnight for fear of contracting COVID-19, as well as the impact of hospital safety measures subsequently leading to restrictions on who could accompany their child to the hospital (n=2).

### Impact of COVID-19 on daily lives and routines

Parents (n=23) and CYP (n=12) reported on the psychological impact of COVID-19 and lockdown; feeling fear, anxiety and isolation. A few parents (n=3) and CYP (n=2) reported missing social engagements which subsequently affected the support they would usually receive. Particular concerns relating to employment/returning to work and education were reported by parents (n=2) and CYP (n=1). Limited access to supplies such as food and medication were a cause of worry for a few parents (n=3) and CYP (n=1). The social/educational development of CYP was impacted due to lockdowns. Parents were challenged in balancing their work commitments and providing home-schooling to their child. Some parents (n=9) and CYP (n=1) reported little to no impact due to COVID-19 on their lives.

### Provision of information and support for parents and children and young people with hydrocephalus

A small number of parents reported receiving mixed messages (n=2) and limited information (n=2) in relation to risks and measures due to COVID-19. A few parents (n=3) reported making decisions about how to support their child using information from charities/government guidelines. Experiences in the provision of support to parents by health professionals varied for parents (n=8) and CYP (n=4), with some respondents expressing ‘great’ support and others reporting ‘little to no support.’ Specific questions that young people and parents reported needing further information related to the risks of contracting COVID as a person with hydrocephalus (n=6, CYP) (n=18, parents). Parents (n=4) and CYP (n=3) also reported questions about shunt related concerns (risks post shunt revision, virus potentially causing shunt blockage, and COVID-19 affecting shunt procedures .) and to how to protect themselves (n=1)/their child post lockdown (n=4).

Responses to the other additional open text boxes relating to information, decision making and support needs further reinforce the themes presented above; for more details see Table S3 (Online Resource 2).

## Discussion

This study aimed to outline the pandemic’s impact on parents and CYP who have hydrocephalus, in order to gain further insight into the experiences and needs of these families during COVID-19. The findings highlighted the significant impact of COVID-19 and lockdown on the wellbeing of parents and CYP with hydrocephalus: reporting feelings of uncertainty and anxiety and particular worries related to the virus itself, how the virus could impact them/their child, about feeling more isolated during the virus outbreak, the impact of cancelled/delayed treatment and if they/their child needed shunt surgery during the pandemic. A study that explored the impacts of the COVID-19 pandemic on healthcare provision and lived experiences of patients with hydrocephalus share similar findings that patients were challenged in accessing treatment in the form of surgery and post-surgical follow up due to COVID-19 [17]. Disruptions to access of healthcare provision and support further challenged the support available to parents and CYP. Furthermore anxiety and stress were further exacerbated by social, educational and financial uncertainties of lockdown. The significant psychological impact of COVID-19 on the mental health of CYP with serious health conditions and parents is evidenced in other studies [3, 4, 9, 18, 19, 25]. There is likely to be a significant long term impact of missed health, education and social encounters for CYP and their families [26, 27].

Parents and CYP reported receiving limited information about the virus or hydrocephalus from their clinical team. Although many parents and CYP used social media for information about the virus; few trusted the advice provided on social media. There was a need for information on how the virus could affect people with hydrocephalus and for communication/reassurance and generally checking-in with CYP and their parents from clinical teams. The importance of proactive communication between parents, CYP and health professionals is highlighted in other studies that explored the impact of the pandemic on children and young people with serious conditions and their parents [4, 9, 18]. Increasing the frequency of communications with CYP and parents may help ameliorate anxiety linked to the stressors, and uncertainty caused by the pandemic, and may also help to mitigate adverse effects of worsening hydrocephalus symptoms by increasing perceived access to support from clinicians [17]. Clinical services had to be adaptive during the pandemic, and improved approaches to remote care via virtual appointments have been evidenced to facilitate high quality care through ease of access to support and providing opportunities for open communication between CYP, parents and clinicians, as well as access to multidisciplinary support across primary, secondary and tertiary care services [28].

Interestingly, not all respondents to the survey reported being negatively impacted by the pandemic. It is unclear whether this may be due to existing support networks from family/friends or clinicians or their access to information that may have alleviated any worries or concerns about the impact of the virus on themselves/their child. The findings from this study are limited to the impacts of the initial few months of the COVID-19 pandemic. Further mixed method studies exploring the longer term impacts of living with hydrocephalus as the pandemic continues would be beneficial. In particular, the impact on the quality of life on CYP with hydrocephalus with different treatment prognoses and varied aetiology of hydrocephalus, and parents who have a child with hydrocephalus. As the pandemic is continually evolving, healthcare and other service providers need to adapt to these changes to ensure the provision of high quality support targeted to those with specific health conditions.

Limitations of this study included the use of digital platforms for recruitment which potentially excluded those without access to digital devices. The majority of parent respondents were mothers and therefore the data is more representative of mothers’ experiences than other types of parent (e.g. father, carer etc.). We also acknowledge that the number of respondents to the survey are low and therefore conclusions need to be interpreted with caution.

## Conclusion

In conclusion, the impact of COVID-19 and experiences of lockdown caused disruption to the lives and routines of CYP with hydrocephalus and parents regarding work, education, social lives, as well as challenged access to treatment and other healthcare support, which led to a subsequent impact on individuals’ psychological and mental wellbeing. The findings identified the need for ongoing, clear and personalised information and communication, particularly in relation to the risks associated with contracting COVID-19 and shunt related concerns; in order to meet the needs and concerns of CYP with hydrocephalus and their families.

## Supplementary Information

Below is the link to the electronic supplementary material.

Supplementary file1 (DOCX 22 KB)

Supplementary file2 (DOCX 15 KB)

## Data Availability

Data available on request from the authors- The data that support the findings of this study are available from the corresponding author upon reasonable request.
