# Supplementary material for: COVID-19- Experiences and support needs of children and young people with Hydrocephalus and parents in the United Kingdom
Source: Childs Nerv Syst. 2023 May 20;39(11):3255–62. doi: 10.1007/s00381-023-05980-7 (PMC10199430; doi:10.1007/s00381-023-05980-7)
Supplement: Supplementary file 1 — Supplementary file1 (DOCX 22 KB) [file 381_2023_5980_MOESM1_ESM.docx]

**TABLE 2. Qualitative findings from open questions under Experiences**

| **Theme** | **Subcodes** | **Number of Quotes** | | **Quotes** | |
| --- | --- | --- | --- | --- | --- |
|  |  | **CYP** | **Parent** | **CYP** | **Parent** |
|  |  |  |  |  |  |
| **Healthcare and treatment provision: delays and challenges to access and availability of care** | Delays in test results & treatment | - | 4 | - | *Routine medical appointments and scans inc. post op MRI and ct been postponed. Therapies cancelled inc. physio, ot and orthotics/splints.*  *Some supply/delivery issues for medication.* |
|  | Cancelled appointments/therapies | - | 9 | - | *[…] because of the virus we have not had the specialists checking on him and monitoring him which is concerning as a lot of the responsibility is with us.* |
|  | Concerns about impact of hospital safety measures & visiting hospital/overnight stays | 2 | 11 | *If I needed to go to hospital I would be worried about COVID-19.* | *In addition to the delay in scanning, there were restrictions on who could accompany children to hospital which meant I had to be alone in hospital for a week whilst she ended up undergoing two shunt revisions.* |
|  | Access to hospital services | - | 3 | - | *He has had hospital appointments cancelled and I am very worried about the effect of this and how difficult it will be to access hospitals with the back log of appointments.* |
|  | Worries about requiring shunt surgery during pandemic/lack of availability of care | 4 | 3 | *My follow up appointment was during the pandemic and was a phone call. Due to this I’ve been worried to go to the doctors as I was discharged without any information or guidance and lots of unanswered questions.*  *Therefore, I have been put off by going to the GP with problems that I have had with my shunt, and worries.* | *I worry that if he has a shunt failure the usual care may not be available.* |
|  |  |  |  |  |  |
| **Impact of COVID-19 on daily lives and routines** | Worries and concerns (psychological impact) | 12 | 23 | *In terms of how the condition affects mental health in general is quite a lot, add in shielding at home and others now being able to see what having a chronic condition is like it does get me down a lot […].* | *Very very isolated, out of routine, spoiled her last year at school which she’s very upset about. I decided not to send her respite to keep her safe as that’s my main priority*  *but it’s resulted in me being very worn down and I think she’s sick of me and her dad.* |
|  | Disruption to family/friend support (missed support/lack of ability to provide support) | 2 | 3 | *Not being able to see family + friends is upsetting.* | *It has ripped our safety net away as parents to not be able to rely on the help of family members.* |
|  | Social/education development of child | 2 | 1 | *I as a young person am very worried about this virus, I have been home for 8 weeks now doing college work from home.* | *she had also just started nursery so I worry about the impact this will have on her education as i am still working full time from home so ensuring that there is enough time in the day to ensure she is still learning and developing is very difficult*  *I am also not a teacher and so cannot pick up if she is struggling or needs more help or whether we need to go through the statement process* |
|  | Concerns about accessing supplies (food/medication) | 1 | 3 | *Other concerns is the lack of delivery service from supermarkets, as I am unable to drive for 6 months after my operation and not being on the shielding list has made this hard. In addition, supermarkets have also made it hard for someone (in my household) to take me shopping as they have limited only 1 person. This makes it hard for people who buy their own groceries but live with other people, especially when you cannot get a delivery slot.* | *Some supply/delivery issues for medication.* |
|  | Returning to work/education | 1 | 2 | *Going back to work worries me although no cases there, keeping 2 metres at work is near impossible.* | *I am worried that once it's time to go back to school and back out in the world that he will become very very anxious.* |
|  | Coping with impact of COVID | 2 | - | *In the beginning I was bored, but now am ok, am doing some drawing, colouring, playing with IPad.* | - |
|  | No/little impact | 1 | 9 | *My experience has been relatively good. I have never had a shunt blockage and I mostly always only go to the hospital for regular check-ups. I don't really have any worries […].* | *My daughter is in good health and no concerns at all during virus* |
|  |  |  |  |  |  |
| **Provision of information and support for parents and CYP with hydrocephalus.** | Decision making using information from charities/government guidelines | - | 3 | - | *[…] Stayed at home and followed government guidelines and advise from shine*. |
|  | Differences between services and health professional support | 4 | 8 | *It has obviously made it harder and longer between appointments in helping with the headache which is really upsetting but completely understandable as doctors are needing to be spread so thinly for everyone during this time.* | *Treatment at hospital and contact with surgical team was great while we were there.*  *Little support since discharge from hospital. Still waiting for appointment dates for MRI scan and to see optometrist. One visit and one video call with Nurse Specialists. It’s been really really difficult, feel in limbo and quite isolated.* |
|  | Consistency of messaging and advice | - | 1 | - | *Mixed messages*. |
|  | Limited information (need for more info) | 1 | 2 | *I would like to given more information on dealing with this virus as a spina Biffida and Hydrocephalus sufferer and know if it does affect me more or not.* | *We were not given any information about any additional risks or additional measures.* |
|  |  |  |  |  |  |
|  |  |  |  |  | |
