# Supplementary material for: COVID-19- Experiences and support needs of children and young people with Hydrocephalus and parents in the United Kingdom
Source: Childs Nerv Syst. 2023 May 20;39(11):3255–62. doi: 10.1007/s00381-023-05980-7 (PMC10199430; doi:10.1007/s00381-023-05980-7)
Supplement: Supplementary file 2 — Supplementary file2 (DOCX 15 KB) [file 381_2023_5980_MOESM2_ESM.docx]

| **TABLE 3: Additional open text boxes** | | |
| --- | --- | --- |
| Questions and Themes | CYP  (N) | Parents (N) |
|  |  |  |
| **Information** | | |
| ***Source of Information*** | | |
| SHINE Charity | 5 | 13 |
| Government (Daily Briefings; Government Website; Letters and texts) | 6 | 23 |
| Hospital (clinical/community team) | 3 | 8 |
| News (TV, newspaper, internet) | 11 | 25 |
| BBC (news/website) | 3 | 4 |
| Social Media (Facebook, Twitter, Instagram) | 2 | 9 |
| Other hydrocephalus or non-hydrocephalus related charities | 1 | 5 |
| Public health organisations (WHO, ONS, NHS) | 4 | 14 |
| Work | 2 | 1 |
| GP | 1 | 1 |
|  |  |  |
| ***Additional Information Required*** | | |
| More information on what is safe to do during pandemic | 1 | - |
| How virus affects individual with hydrocephalus | 1 | - |
| Protecting themselves/their child post COVID (incl. return to work) | 1 | 4 |
| Impact on further education | 1 | - |
| Post-operative risk if COVID contracted | - | 1 |
| Shunt related issues | 3 | 4 |
|  |  |  |
| **Decision Making** | | |
| SHINE | 2 | 1 |
| Medical/health team (incl. GP) | 3 | 5 |
| Government (incl. guidelines on shielding/letter/isolating) | 2 | 11 |
| Common sense/intuition | - | 3 |
| Own decisions/knowledge | 3 | 10 |
| Weighing up risks and benefits | 1 | 3 |
| Partner/family members | 3 | 1 |
| Scientific Literature | 3 | 2 |
| Previous experience of managing condition/personal circumstances | 2 | 2 |
| Other leading health bodies (PHE, WHO) | - | 2 |
| Other charities | 1 | - |
|  |  |  |
| **Support** | | |
| No additional support required | 7 | 13 |
| More relevant/clear information/more guidelines (for those shielding/ how it affects those with hydrocephalus/dandy walker malformation) | 6 | 18 |
| Mental Health Support (for CYP) | 1 | - |
| Psychological support for parents | - | 1 |
| Support from Hospital (check in/contact/reassurance from clinical team) | 1 | 12 |
| Support for parents (speaking to other parents, help whilst shielding) | - | 4 |
| Financial/Employment/Education support | 1 | 3 |
| COVID-19 testing/priority of testing | 1 | - |
|  |  |  |
|  |  |  |
| N=number of quotes; Numbers do not correspond with the ones provided in the section Qualitative Findings as comments were often broken down and categorized under a number of different themes. | | |
